# Towards a Conceptual Model of Diabetes Self-Management among Chinese Immigrants in the United States

**DOI:** 10.3390/ijerph110706727

**Published:** 2014-06-27

**Authors:** Bin Zeng, Wenjie Sun, Rebecca A. Gary, Changwei Li, Tingting Liu

**Affiliations:** 1 Neurosurgical Intensive Care Unit, Sichuan Provincial People’s Hospital, Chengdu 610072, China; E-Mail: zengbin138@sohu.com; 2 School of Food Science, Guangdong Pharmaceutical University, Zhongshan 528458, China; E-Mail: sunwenjie2002@hotmail.com; 3 School of Public Health and Tropical Medicine, Tulane University, New Orleans, LA 70112, USA; E-Mail: cli8@tulane.edu; 4 Nell Hodgson Woodruff School of Nursing, Emory University, Atlanta, GA 30322, USA; E-Mail: ragary@emory.edu

**Keywords:** Chinese immigrants, diabetes self-management, transcultural health, conceptual model, type 2 diabetes

## Abstract

*Background*: Chinese immigrants have been disproportionally affected by type 2 diabetes. This paper presents the state of science regarding the factors that may influence diabetes self-management among Chinese immigrants in the US and the potential health outcomes. *Design*: Using Walker and Avant’s techniques, a search of the literature was conducted from CINAHL, PubMed, OVID, and Web of Science. *Findings*: Factors most relevant to diabetes self-management were grouped under five categories: socio-demographic characteristics, behavioral and psychological characteristics, social support, linguistic barriers, and cultural characteristics. Potential outcomes derived from improved diabetes self-management include quality of life, glycosylated hemoglobin, and blood pressure and other cardiovascular risk factors. *Discussion*: A conceptual model was provided to guide future research. Based on the review of the literature, specific research topics that need to fill the gaps in the literature were provided, including family-focused interventions for Chinese immigrant patients with diabetes and the effectiveness of these interventions to improve family functioning.

## 1. Introduction

The World Health Organization projects that worldwide more than 180 million people have diabetes, and estimates this number will double by the year 2030 [1]. Despite reports of less obesity and a lower mean body weight than non-Hispanic whites in the United States (U.S.), both the incidence and prevalence rates of type 2 diabetes mellitus (T2DM) have increased dramatically among Chinese immigrants (CIs) [2], but the reason is not well understood [2,3]. Length of residence in the U.S., however, was found to contribute to the high prevalence of T2DM among immigrants in general [3]. As the population of CIs with T2DM continue to rise, it is important that health care providers caring for this population develop optimal strategies to guide T2DM self-management.

Self-management has been referred to as a set of daily behaviors that patients perform to manage their diabetes [4]. Effective self-management for T2DM is important for improving diabetes related health outcomes [5]. Although a framework for self-management of chronic conditions has been developed [5], a culturally appropriate theoretical model is needed to guide T2DM self-management for CIs, since many continue to believe in and practice Traditional Chinese Medicine (TCM) after immigrating to the U.S. [6,7]. The conceptual model presented in this paper will build upon previous studies of T2DM self-management in CIs as well as recommendations to guide future studies. The purpose of this article, therefore, is to: (a) examine the existing literature related to T2DM self-management, (b) examine the relationship of T2DM self-management to health outcomes in CIs, and (c) propose a conceptual model for enhancing self-management for T2DM in CIs. Since limited studies have been conducted among CIs with T2DM, the conceptual model presented in this paper can be considered as an initial step towards a theory of T2DM self-management in CIs with T2DM.

## 2. Background

The U.S. Office of Management and Budget defines Asians as people having origins in any of the original people of the Southeast Asia, Far East, or the Indian subcontinent, including Cambodia, China, India, Japan, Korea, Malaysia, Pakistan, the Philippine Islands, Thailand, and Vietnam [8]. The increasing number of Asian immigrants in the U.S. has changed the composition of U.S. population [9]. The Asian population increased by 43% from 10.2 million in 2000 to 14.7 million in 2010, and by 2050, there will be more than 40.6 million Asians living in the U.S., comprising 9.2% of the total U.S. population [8]. T2DM is one of the most debilitating diseases in the U.S., with an estimated prevalence of 10.7% among all adults aged 20 years and older [10]. Diabetes affects about 10% of Asians, with the majority developing T2DM [11]. Asians have a greater risk for developing T2DM compared to Whites, particularly for those who are foreign born and those with normal weight [11,12,13]. As the largest Asian subgroup (3.8 million) in the U.S. [8], CIs have been disproportionately affected by T2DM, as the incidence of T2DM rose to 14.7% [2], exceeding the average prevalence rate (10.7%) among U.S. population and Hispanics (10.4%) [14], although still lower than that (18.7%) of T2DM among African Americans [15]. The increase in incidence of T2DM among CIs may be attributed to a combination of genetic and environmental factors [16]. However, such an increase occurred within a relatively short period of several decades, indicating environmental and behavioral changes may play a greater role than genetic factors [17].

Despite the burgeoning growth of the CIs in the U.S. [8], their health needs are not well met and it is becoming an increasingly visible problem as prevalence of certain chronic illnesses, including T2DM, continue to rise [18]. CIs and other Asians are often not enrolled in T2DM studies and other lines of health research for a variety of reasons, so little is known about chronic illness in this population [18,19]. Additionally, the tendency to study ethnicity primarily as Black and White further obscure the health needs of CIs [19]. The demographic trend of large numbers of CIs’ immigrating into U.S. over the past several decades indicates that the number of CIs affected by T2DM is likely to continue. Despite this projected increase, there is little documented diabetes-related research exclusively on CIs, indicating an urgent need for research on T2DM self-management in this growing population.

Conceptual models provide a blueprint for understanding a specific phenomenon. Conceptual model development also provides a mechanism to identify and express central ideas about the essence of health care practice [20]. Although there are a number of strategies for conceptual model development, Walker and Avant’s 3-step methodology [20] was used to guide conceptual model development. According to Walker and Avant [20], procedures of theory synthesis include identification of focal concepts, identification of relationships of concepts, and construction of an integrated representation. 

## 3. Experimental Section

### 3.1. Data Sources

Following the Walker-Avant methodology, an extensive search of the literature was conducted. Literature retrieval was conducted using the following databases: CINAHL, PubMed, OVID, and Web of Science. The following terms were entered for the first round of literature search as key terms for the related journal articles published from 2004 to 2014 and included: diabetes mellitus, type 2, self-care, and Chinese immigrants. Since self-care and self-management, and Chinese Americans and Chinese immigrants have been used interchangeably in the literature, diabetes mellitus, type 2, self-management, and Chinese Americans were also entered during second round of search in order to obtain a full list of relevant research reports. The electronic search was supplemented by a manual search of current issues of periodicals and follow-up of other cited materials. Identified duplicates among these databases were removed.

### 3.2. Search Outcomes

First, the search terms “self-care”, “self-management”, “diabetes mellitus, type 2”, and “Chinese Americans” were entered, locating 42 journal articles from four databases. Then, 13 duplicates were removed, and 29 articles were remained for abstract review. After careful content reading, eight articles were excluded because these studies were carried out in places other than the U.S. or in other populations. In addition, one article was identified as relevant by hand searching reference lists of retrieved articles or key journals. A total of 22 journal articles were downloaded for full-text review, including 10 descriptive studies, one pilot study, two quasi-experimental studies, and nine qualitative studies. To date, no randomized controlled trials have been reported exclusively in CIs with T2DM. Based on the variables reported in these articles and the relationships with T2DM self-management, those variables most relevant to T2DM self-management were broadly grouped together under five categories: socio-demographic characteristics, behavioral and psychological characteristics, social support, linguistic barriers, and cultural characteristics. These categories are identified in the conceptual model as related factors that may influence T2DM self-management among CIs. Potential outcomes derived from improved T2DM self-management include quality of life (QOL), HbA1c, blood pressure and other cardiovascular risk factors. 

## 4. Results

### 4.1. The Conceptual Model

**Figure 1:**
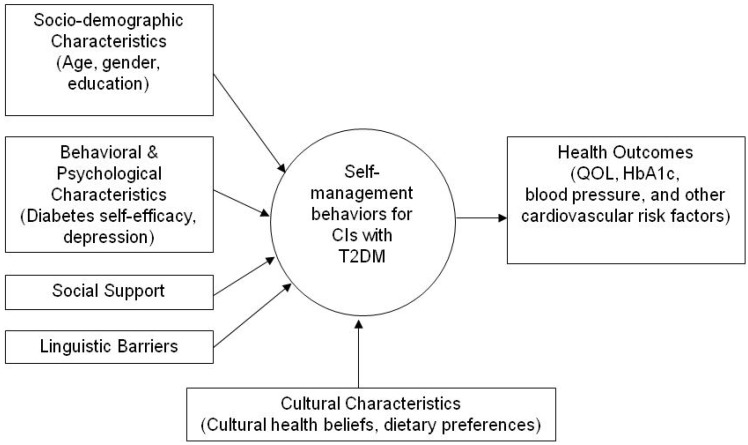
Towards A Conceptual Model of T2DM Self-Management in Chinese Immigrants with Type 2 Diabetes Mellitus.

The model is illustrated in Figure 1. It provides a comprehensive framework to explore the relationships that influence T2DM self-management among CIs. 

Seven key interrelated components comprise the model and include: socio-demographic characteristics, behavioral and psychological characteristics, social support, linguistic barriers, cultural characteristics, T2DM self-management, and health outcomes. This model postulates that socio-demographic characteristics, behavioral and psychological characteristics, social support, linguistic barriers, and cultural characteristics impact T2DM self-management. These factors together then predict health outcomes. In Chinese culture, strong family bonds are important and highly valued [21]. Medical treatment and decision should be made with family members rather than by the discretion of the individual [22]. Thus, the model also assumes that T2DM self-management primarily takes place within a family context, and both individuals and family members are influenced by the Chinese culture. Walker and Avant suggested that conceptual model development begins with identification of focal concepts [20]. A focal concept is the starting point from which a theorist moves out to other interrelated variables or concepts [20]. T2DM self-management was identified as the focal concept in the proposed conceptual model because it is key for improved health outcomes in CIs. 

#### 4.1.1. T2DM Self-Management

Based on studies to date of non-CIs, T2DM self-management has been found to be critical to achieve improved clinical outcomes in T2DM [23]. A meta-analysis of self-management interventions for T2DM revealed that self-management interventions can improve glycemic control [24]. T2DM self-management behaviors include dietary modification, foot care, medication adherence, oral hypoglycemic agent administration, physical activity, glucose monitoring, and coping with the side-effects of the drug and illness progression. Since self-management involves complex and comprehensive skills, patients with T2DM must learn to evaluate and integrate a variety of these activities into their daily life to successfully perform self-management. Most T2DM treatment relies on the ability of patients to perform these self-management behaviors correctly. Studies of T2DM self-management across racial and ethnic groups document the group’s culture and social networks can directly influence self-management behaviors [16]. Culture and social networks affect people’s ways of thinking, which people use to inform their health behaviors.

Followed by identifying focal concept of self-management, a careful search and comprehensive literature was conducted to identify related factors [20]. The nature of the relationships, including the direction and the strength of relationships were also identified for T2DM self-management behaviors. Based on the relationships with self-management, these relationships will be presented in details under the subtitles of socio-demographic characteristics, behavioral and psychological characteristics, social support, linguistic barriers, and cultural characteristics. Finally, as the last step in Walker and Avant’s 3-step model [20], the construction of an integrated representation is presented in Figure 1 above. 

#### 4.1.2. Socio-Demographic Characteristics

##### Age

Two studies found that age was positively associated with T2DM self-management. For example, in one study, the positive coefficient between age and T2DM self-management was 0.33 (*p* < 0.05), indicating older CIs were more likely to perform T2DM self-management than younger CIs [25]. In terms of specific T2DM self-management behaviors, older CIs were more likely to follow dietary modification (odds ratio = 1.04, *p* < 0.05), take exercise (odds ratio = 1.04, *p* < 0.05), and carry out foot care (odds ratio = 1.04, *p* < 0.05) compared to younger CIs [18]. A possible explanation for this finding is that young people who are employed could be busy, making it difficult to better manage T2DM [18]. However, the strength of the association between older age and specific T2DM self-management behaviors seems weak.

##### Gender

The research on the effect of gender on T2DM self-management among CIs has yielded inconsistent findings. In the most recent study of T2DM self-management among CIs, gender did not have significant relationships with any aspects of T2DM self-management behaviors, including medication taking, dietary modification, exercise, and glucose monitoring [18]. In another study, CI women were more likely to engage in T2DM self-management practice than the male counterparts [25]. The inconsistency in the literature regarding the role gender may have on T2DM self-management behaviors warrant further investigation.

##### Education

Variations in self-management practice were found by educational level among CIs. For example, in the most recent study of T2DM self-management, higher educational level has been linked to better self-management in terms of taking exercise and glucose monitoring [18]. It was speculated that people with low educational level might have difficulty in understanding diabetes-related information regarding lifestyle changes and glucose monitoring [18]. However, CIs with higher educational level were found to be less likely to take medication (odds ratio = 0.64, *p* < 0.05) and maintain foot care (odds ratio = 0.75) [18]. The underlying reason for these relationships is not well understood. 

#### 4.1.3. Behavioral and Psychological Characteristics

##### Diabetes Self-Efficacy

Coined by Bandura in 1977, self-efficacy is a key tenet in the Social Cognitive Theory [26]. Diabetes self-efficacy has been defined as the judgment of one’s own capability to monitor, plan, and perform diabetes activities [27]. One study found that T2DM self-management was significantly and positively correlated with efficacy expectations (*r* = 0.54) and outcome expectations (*r* = 0.44) in 145 patients with T2DM in Taiwan [28]. Self-efficacy has been reported to be associated with T2DM self-management in ethnically diverse populations as well. In a study with a diverse ethnic sample (18% Asian/Pacific Islander, 25% African American, 42% Latino/a, and 15% White), Sarker and associates concluded that higher self-efficacy score was associated with improved T2DM self-management behaviors, across both race/ethnicity and health literacy levels [29]. These findings support that incorporating self-efficacy into a T2DM education program may be a strategy to enhance T2DM self-management and related health outcomes among CIs.

The definition of self-efficacy indicates that self-efficacy is behavior-specific, and efficacy beliefs may vary considerably across health behaviors [30]. For instance, if an individual has high self-efficacy with regard to exercise, this may or may not be generalized to persistence in diet management. Glucose monitoring self-efficacy, exercise self-efficacy, dietary self-efficacy, and medication self-efficacy have been separately evaluated and correlated with the frequency of adherence to glucose monitoring, exercise, diet, and taking oral medication, respectively. The Pearson’s correlation coefficients were reported as 0.47, 0.67, 0.50, and 0.25, respectively [31]. Therefore, understanding T2DM self-efficacy is complicated because performance in one field does not necessarily predict performance in another. It has been suggested that self-efficacy should be evaluated and presented separately for each aspect of self-management behaviors rather than constructing a general measure of self-efficacy without reference to the specific behavior and situation [31]. However, it is not clear what component of self-efficacy would have the greatest impact for improving self-management of T2DM in CIs.

##### Depression

Depression is particularly common in patients with T2DM. The prevalence of depression was significantly higher among individuals with T2DM (17.6%) compared to those without T2DM (9.8%) [32]. The authors also reported that prevalence of depression was higher among females with diabetes (23.8%) compared with their male counterparts (12.8%). Growing evidence has consistently demonstrated that depressive symptoms is associated with worse T2DM self-management practice with respect to taking medication, dietary modification, and exercise [33,34,35]. Depressive disorders may also place T2DM patients at increased risk for long-term complications such as poor adherence to medications and other self-care regimens [35]. Currently, the reason for these associations remains largely unknown since cross-sectional data were used for all these studies. In addition, compared to their White counterparts, CIs may be less likely to seek help from health care providers due to the stigma associated with mental illness in Chinese culture [36,37]. Mental illness is generally viewed as degrading not only to the patient, but also to the entire family. For this reason, they may feel ashamed to seek or participate in prescribed treatments. Therefore, CIs with T2DM are more likely for their depression to go undetected and remain untreated, which could adversely limit their ability perform effective T2DM self-management.

#### 4.1.4. Social Support

Social support is defined as an interaction involving two or more people whose purpose is to provide awareness and education, provide emotional instrumental, financial support, and assist with problem-solving skills [38]. Gallant [39] provided evidence of a modest positive relationship (0.09 to 0.49) between social support and chronic illness self-management, especially for T2DM. A systematic review of randomized controlled trials literature on the effect of the social support focused interventions on self-management and health outcomes in T2DM found that based on limited evidence available, social support is influential on self-management and outcomes of T2DM [40]. General support from one’s family is related to improved subjective health [41], while family separation undermined T2DM management in CIs [42]. In particular, patient-appraised marital satisfaction was independently linked to T2DM management [43], because high relationship satisfaction between spouse and patient would most likely create a positive emotional atmosphere for support and empathy [44].

Different sources of social support have been linked to specific T2DM self-management behavior. Support from family and friends had a beneficial effect on glucose monitoring, foot care, and following a diabetes meal plan, whereas support from health care professionals was negatively associated with adherence to a diabetes meal plan among African-American and Latino diabetic patients [45]. Likewise, Shaw and colleagues reported that support from family and friends was positively associated with adherence to diabetes meal plan and foot care, and support from neighborhood was positively related to adherence to diabetes meal plan, foot care, and physical activity [46]. Support from community was not related to any of self-management behaviors [46]. These studies suggest that one source of social support may not necessarily improve all self-management behaviors and related health outcomes. However, to date, no specific study has examined the relationship between social support and T2DM self-management in CIs. Further research is especially needed to address the following questions: Which source of social support is linked to which specific self-management behaviors in CIs? 

#### 4.1.5. Linguistic Barriers

Linguistic barriers have been repeatedly reported to result in communication problems with health care providers among CIs which leads to misinterpretation of information and poor diabetes outcomes [16,42,47]. CIs who preferred to speak English demonstrated higher levels of diabetes knowledge and lower level of HbA1c than those preferred to speak Chinese [47]. CIs who spoke English were also linked to receiving more T2DM self-management advice from health care providers [48]. In addition, linguistic barriers also compromised CIs’ ability to schedule an appointment and purchasing over-the-counter diabetes care products (e.g., glucose monitors) [42]. Since printed T2DM education materials are scarce in Chinese in the U.S., CIs may be less likely to retain critical information that enhances diabetes-specific knowledge [6,47], thereby adversely influencing T2DM self-management behaviors. Focus group discussions with CIs revealed that employing culturally competent health care providers or providing professional interpreter service might increase their ability to identify learning issues and promote better self-management [47,49]. However, it is worth noting that cultural familiarity and competency goes much deeper than language proficiency and simple translation. Although CIs found Chinese-language diabetes educational materials easy to read and comprehend, some CIs still thought that the verbatim translated diabetes educational materials were too simplistic to be useful. For example, there were no dietary recommendations for Chinese foods [42]. Therefore, providing language translation service is only the first step in achieving better diabetes care. More importantly, cultural knowledge on a specific area of diabetes care can also affect diabetes care. 

#### 4.1.6. Cultural Characteristics

##### Cultural Health Beliefs

Culture influences an individual’s health beliefs and attitudes, thereby impinging on T2DM self-management behavior [50]. According to Andrews and Boyle, there are three views of health beliefs: magico-religious, holistic, and scientific [51]. The holistic health paradigm, which is very common in Chinese culture, attributes illness to natural imbalance. The goal of treatment is therefore to re-achieve yin-yang balance through a holistic approach [52]. The holistic health paradigm is contradictory to the western biomedical model, which takes a disease-specific focus [53]. 

Because the theories of TCM are based on thousands of years of experience, TCM is still commonly practiced in China and among CIs is the U.S. [25,54]. On the one hand, the strong adherence to traditional Chinese health beliefs among CIs with T2DM is evidenced by their reluctance to using Western medicine and desire to incorporate TCM into their diabetes treatment regimen [6]. For example, CIs with T2DM tried to maintain a food balance in their diet management. The central idea embedded in the food balance is that certain foods have a “hot” property while others have a “cold” quality based on TCM [53]. On the other hand, extent to which CIs with T2DM seek cultural health treatments is contingent upon the level of acculturation of the patient and the family [19]. During acculturation, CIs are confronted with multiple demands in terms of accessing health care and evaluating different treatment options. They may respond by sticking to their cultural health beliefs, or adopting new disease management strategies, or integrating different forms of treatment plan together [19]. The practice of Western medicine, Chinese holistic treatment, or treatment combination of both approaches suggests a strong effect that health beliefs and practices have on T2DM self-management among CIs. Those who are less acculturated may choose to treat T2DM with TCM to restore yin-yang balance. However, the differences on how Western medicine or TCM treatment plan influences T2DM self-management remains unclear and warrants further study. Further research is also needed to address how acculturation affects T2DM self-management and health seeking patterns in CIs. 

##### Dietary Preferences

Cultural factors to consider in T2DM self-management should also include dietary preferences, which may present a great challenge in managing T2DM. In general, foods have been conferred an important and special cultural meaning in traditional Chinese culture: they are not only considered as a survival need, but also the freedom to enjoy foods plays an integral part of people’s quality of life [19]. The T2DM dietary restrictions conflict with this cultural norm in that CIs may view these restrictions as a way of preventing them from living a full life [19]. Foods in Chinese culture also serve the purpose of building and solidifying relationships with others in a social setting, and keeping portion sizes in check may diminish the enjoyment and raise concern on how to cater to the desires of others [19]. Overall, it is difficult to resist overeating in a social setting. Additionally, the carbohydrate limit, such as restriction on rice, is particularly challenging [53,55]. In Chinese families, rice is served in almost every meal and is believed to maintain holistic well-being [53]. Reduced rice consumption in a diabetic diet is thus distressing and a source of suffering for CIs with T2DM [53]. Since many CIs are still consuming Chinese foods after they immigrate to the U.S. [56], consultation with dietitians who are familiar with Chinese food preferences and can offer suggestions on culturally acceptable rice substitutes that may ease the transition is warranted [53]. The cultural values about food restrictions underscore the importance of tailoring treatment protocols that should be culturally acceptable and followed by CIs without sacrificing their desire for a full and healthy life. Failure to do so may lead to poor health outcomes for this patient group. In line with the findings of Chesla *et al.*’s [53] study, Hsu *et al.* [47] also reported that CIs who were limited English proficient found it helpful in reading the Chinese diabetes guide which was written in accordance with their cultural dietary practice. Therefore, providing language translation service and cultural knowledge on food preparation is more helpful than providing language assistance alone.

#### 4.1.7. Health Outcomes

##### QOL

One of the important goals for T2DM self-management is to achieve highest possible QOL for the patients [57]. Research on T2DM self-management and QOL has yielded mixed results. Several studies, including a meta-analysis 20 research reports, have shown effective T2DM self-management can lead to better QOL in different ethnic groups, including Taiwanese [58,59,60]. Two studies have reported the change in QOL among CIs after a culturally tailored T2DM educational program [61,62]. However, in one study, the authors did not report how much changes in QOL were achieved in their program [61]. In another study, the authors reported benefits of culturally adapted coping skills training program were evident in improvement in QOL among CIs with T2DM [62]. 

##### HbA1c

HbA1c is considered the gold standard to evaluate how patients have controlled their diabetes in the preceding two to three months. To achieve optimal goals of self-management and reduction of complications in T2DM, adequate glycemic control (*i.e.*, HbA1c < 7%) is required [63,64]. Numerous studies have found that good glycemic control is associated with effective T2DM self-management [65,66,67,68]. Two recent studies reported that CIs improved glycemic control after culturally tailored T2DM self-management program [61,69]. However, both studies employed a sing-group pre-post-test design with a relatively short follow-up period of time. In another quasi-experimental study by Chesla and coworkers [62], there was no significant change in HbA1c after the culturally adapted coping skills training. Randomized controlled trials with longer follow-up evaluation period are needed to test the long-term effect of a culturally tailored T2DM self-management program on glycemic control among CIs. 

##### Blood Pressure and Other Cardiovascular Risk Factors

A number of risk factors for cardiovascular disease have been identified, including diabetes, smoking, and obesity. Typically, the incidence of hypertension and cardiovascular disease increases in patients with diabetes. This is especially true for patients with T2DM [70]. Therefore, blood pressure is used as one of the biological outcomes to assess the risk of CIs to develop secondary cardiovascular disease in this conceptual model. It was one of the key outcomes in the Wang and Chan’s [61] pilot study who developed a culturally-tailored diabetes education program focused on T2DM self-management behaviors. In this study, the participants’ blood pressure decreased and remained within optimal range 3 months after the program indicating that blood pressure may be a highly relevant outcome to explore as part of T2DM self-management. However, it is not clear what changes in other cardiovascular risk factors, such as dyslipidemia, can be made in response to better T2DM self-management behaviors in this population.

## 5. Discussion

The conceptual model presented serves as a beginning approach for a better understanding, development, and testing of T2DM self-management interventions for CIs. Using a culturally focused model that identifies the key variables that influence T2DM self-management and potential outcomes from effective management, may provide a guide for future interventions. Suggested research topics for future investigations that are grounded in current literature and bridge the gaps in knowledge are presented at the end of the description of the variables. Of particular concern is the impact of cultural characteristics on T2DM self-management in CIs. So far, no quantitative study has addressed this issue. Therefore, research in this area should be considered as the most urgent and immediate priority. 

Our study has limitations. First of all, this is not a systematic/integrative review. However, according to the Walker-Avant methodology [20], it does not require a systematic/integrative review when performing conceptual model development. We conducted an extensive literature review, and hence, the presented model is only tentative and may be subject to future modifications. In addition, our extensive literature review has demonstrated where knowledge is lacking, which can then be used to guide future research. In turn, the knowledge gained from future research will help modify the current conceptual model. Secondly, there have been no randomized controlled trials exclusively in CIs with T2DM. The relationships reported in this model should be tested and verified by future clinical trials. 

Based on the review of the literature, the following conclusions can be drawn. First, more attention should be directed to family-focused interventions for CI patients with T2DM. There are a number of personal characteristics that are difficult to modify, including gender and linguistic barriers. Therefore, interventions that focus solely on personal characteristics may show little promise for successful T2DM self-management. The effectiveness of interventions to improve family functioning should be tested for the impact on enhancing T2DM self-management in CIs. Secondly, after all important factors associated with T2DM self-management and outcomes have been identified, a comprehensive approach which integrates personal, family, and cultural characteristics should be taken to develop an intervention study. The conceptual model presented provides an important step to guide T2DM self-management in CIs. The predictors of the T2DM self-management and its associated outcomes are an important area to explore. The gap in the literature is problematic for the purpose of tailoring a T2DM self-management intervention for CIs. With limited resources and inadequate utilization of health care service, T2DM self-management becomes a notably big challenge for them. Although the area of T2DM self-management in CIs is emerging, more research is needed to describe the family barriers to better self-care, to identify the predictors for effective self-management, and to test a culturally-appropriate randomized controlled trial that enhance T2DM self-management. Investigators can apply this conceptual model to locate the most important determinants, which will be the first step to develop and test a much-needed self-management intervention study for this disadvantaged group of patients.

## 6. Conclusion

Overall, the rate of increase in T2DM incidence among CIs in the U.S. will continue to accelerate, in part because of the immigration experience and the burgeoning growth of the CIs in the past several decades. T2DM self-management is therefore important for CIs to achieve better diabetes related health outcomes. T2DM self-management behaviors are influenced by multiple factors, including socio-demographic characteristics, behavioral and psychological characteristics, social support, linguistic barriers, and cultural characteristics. The relationships reported in this conceptual model should be tested and verified by future clinical trials. In addition, more research is also needed to identify the interactions between these factors. The presented conceptual model can serve as a starting point for developing a culturally tailored T2DM self-management randomized controlled trial in CIs. 
